# An Unusual Cause of Abdominal Pain in a Middle-Aged Female: The Nutcracker Syndrome

**DOI:** 10.7759/cureus.30696

**Published:** 2022-10-26

**Authors:** Megan Mary John, Than Zaw Oo, Syed Aftab

**Affiliations:** 1 Acute Care and Emergency Department, Khoo Teck Puat Hospital, Singapore, SGP; 2 General Medicine Department, Sengkang General Hospital, Singapore, SGP; 3 Radiology Department, Sengkang General Hospital, Singapore, SGP

**Keywords:** recurrent abdominal pain, left renal vein compression, lower abdominal pain, non specific abdominal pain, renal nutcracker syndrome

## Abstract

A middle-aged female presented with lower abdominal pain associated with nausea and vomiting and was admitted to the acute hospital. She was thoroughly investigated and treated presumably as having a urinary tract infection. However, she was admitted again shortly after discharge with persistent symptoms. A careful evaluation and review were done, and she was diagnosed with nutcracker syndrome based on the clinical assessment, computed tomography (CT), and ultrasound (US) findings.

## Introduction

To evaluate abdominal pain, a broad differential of common causes, understanding pain mechanisms, and recognizing classical patterns and clinical presentations are essential. The causes of acute lower abdominal pain in women commonly include gynecological, gastrointestinal, and urological diseases. Rarely, neurological and vascular disorders may also occur. When common and life-threatening causes have been excluded, one must pursue less common diagnoses if symptoms persist. Nutcracker syndrome is a rare vascular disorder that describes the compression of left renal vein between the superior mesenteric artery and the aorta [1]. It is a term reserved for patients with corresponding clinical signs and symptoms, such as flank pain, hematuria and pelvic congestion in females, [2] alongside diagnostic imaging of the anatomy associated with the syndrome [3]. In this case discussion, we describe a rare case of pelvic congestion syndrome secondary to the nutcracker phenomenon as a cause of lower abdominal pain in a female of reproductive age.

## Case presentation

A 30-year-old domestic worker from Indonesia (gravida 2, para 2) initially presented with a two-week history of bilateral adnexal pain radiating to bilateral flanks associated with multiple episodes of nausea and vomiting. She was otherwise afebrile and denied any urinary symptoms such as hematuria, abnormal menstrual bleeding, other gastrointestinal symptoms, or a history of trauma. Physical examination revealed tenderness over bilateral flanks, iliac fossae, and suprapubic region. Bilateral lower extremities demonstrated normal strength and sensation with normal pulses, absent varicosities, and no edema.

Blood tests, including full blood count, renal panel, and liver function tests were unremarkable, urine human chorionic gonadotropin (HCG) was negative, urinalysis was clear, and urine cultures showed mixed bacterial growth. In addition, an initial computed tomography (CT) abdomen and pelvis were done, and it showed no definite acute infectious, inflammatory or neoplastic process was appreciated.

**Figure 1:**
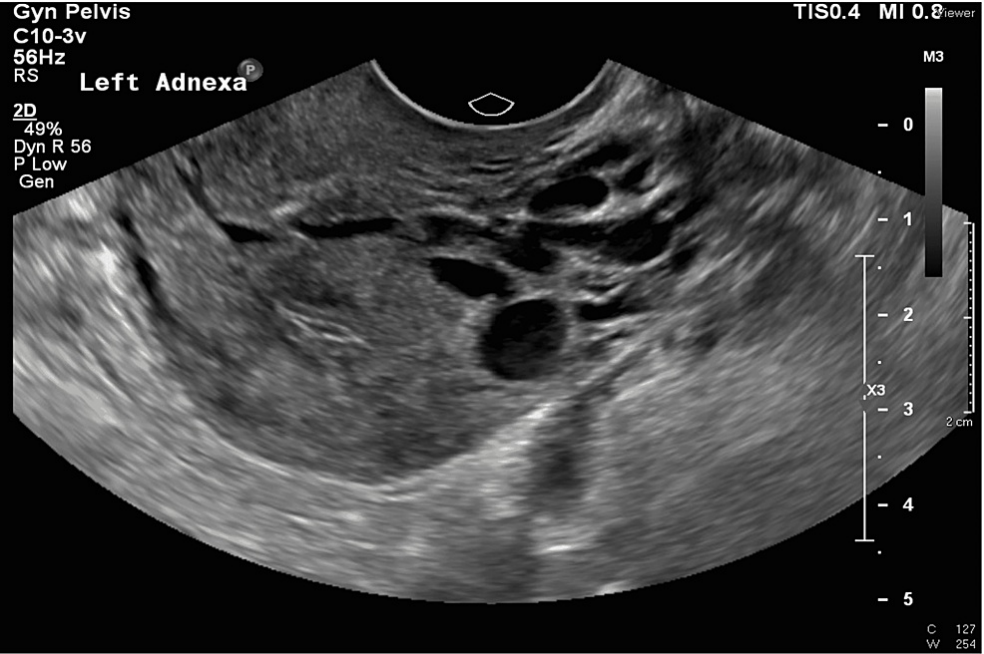
Pelvic ultrasound greyscale demonstrates dilated left adnexal veins

**Figure 2:**
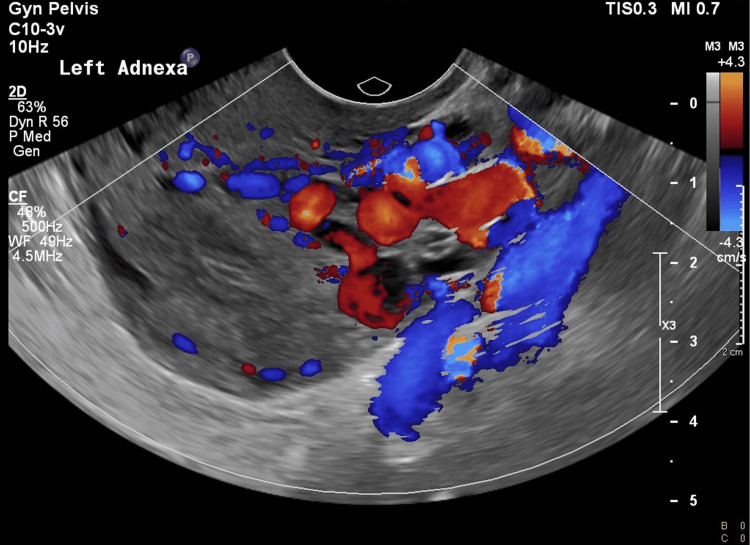
Pelvic ultrasound with color Doppler demonstrates dilated left adnexal veins

Inflammatory markers and total white blood cells were not raised. However, as the patient still had left adnexal pain, an ultrasound (US) pelvis scan was performed, and it displayed dilated and tortuous adnexal veins over the left, suggestive of pelvic congestion syndrome (Figures 1, 2).

The patient was presumptively treated for a urinary tract infection with oral ciprofloxacin over five days, and symptomatic medications for pain control. She was subsequently discharged.

However, four days later, the patient returned with similar complaints with minimal resolution of symptoms. An abdominal X-ray was obtained initially to rule out urinary calculi, given the location and characteristics of the patient’s pain, which revealed only fecal loading. However, there was no resolution of symptoms despite clearing the bowels.

**Figure 3:**
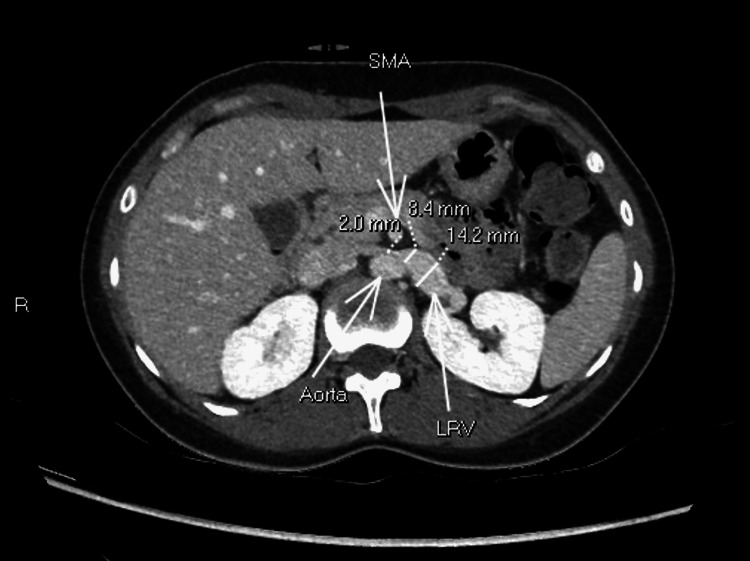
Axial CT images reveal a markedly narrowed LRV (> 50 %) between the aorta and SMA. Pre-stenotic dilatation of the left renal vein is also seen. LRV: Left renal vein, SMA: Superior mesenteric artery

**Figure 4:**
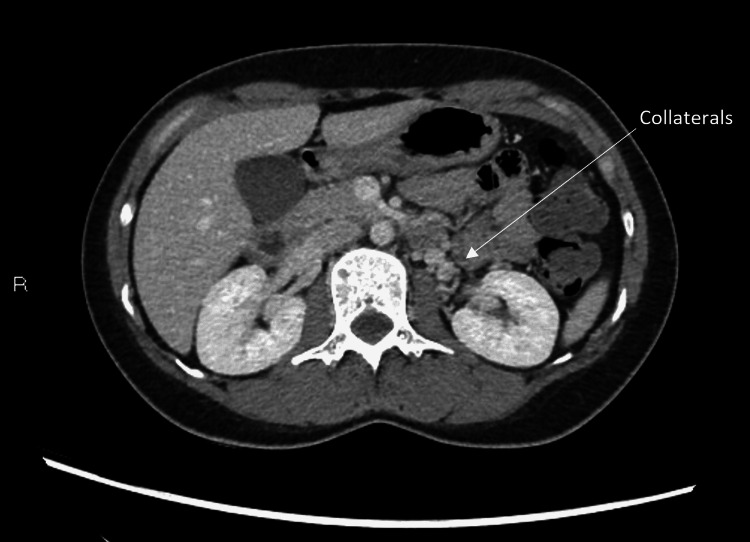
Axial CT images reveal multiple perinephric venous collaterals around the left kidney

**Figure 5:**
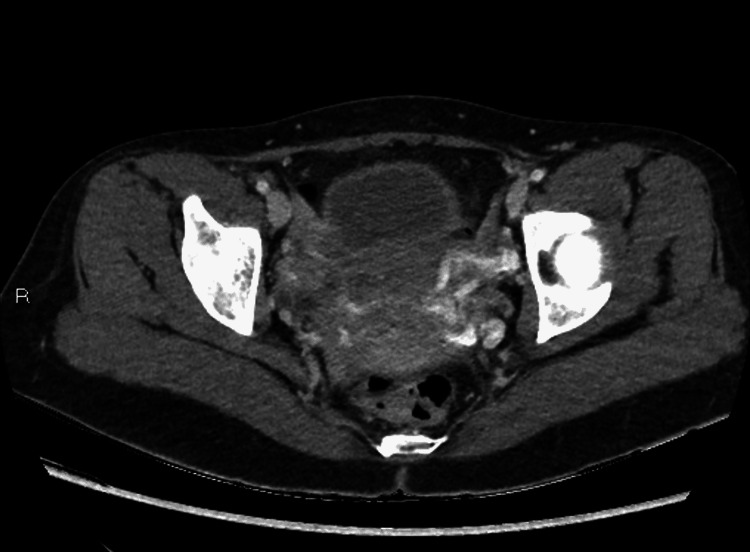
Axial images of the pelvic CT demonstrate asymmetrically dilated left adnexal/pelvic veins

**Figure 6:**
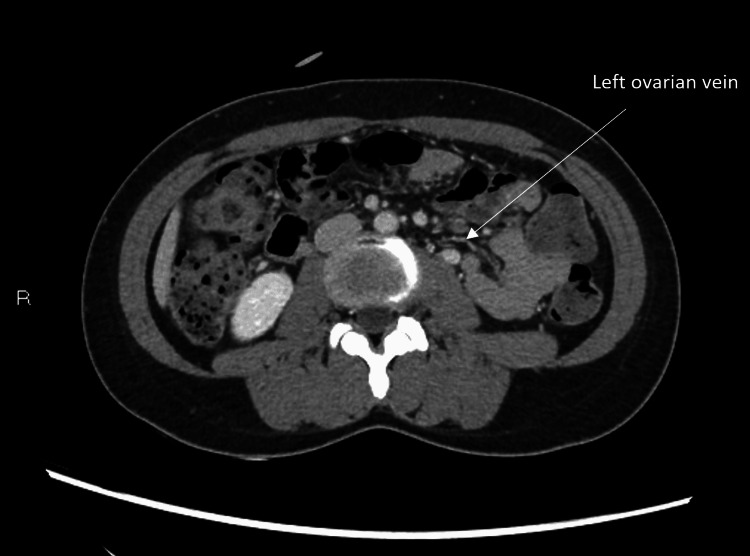
Axial CT images showing asymmetrically dilated left ovarian vein

Given the persistent symptoms and previous findings on the pelvic US, a repeat CT of the abdomen and pelvis was done, which displayed that there was narrowing/stenosis of the left renal vein from the overlying superior mesenteric artery with pre-stenotic dilatation and features of nutcracker anatomy without evidence of intestinal obstruction or suspicious mass (Figure 3). This was associated with early enhancement of the left gonadal vein as well as left juxtarenal and paraovarian collaterals (Figure 4). Moreover, the CT revealed asymmetrically dilated left adnexal/pelvic veins again (Figures 5, 6). Based on these latest CT and US findings, the diagnosis of nutcracker syndrome was made.

Our patient remained hospitalized for nine days for pain control and evaluation for vascular surgery. The option of endovascular stenting was offered to the patient. However, because of financial constraints on our patient’s work permit status, she opted not to proceed with further imaging and interventions during admission. Hence, she was given non-steroidal anti-inflammatory drug (NSAID) analgesia and treated with conservative management and discharged with outpatient appointments for further discussions on vascular surgery and obstetrics and gynecology. Unfortunately, the patient did not turn up for the subsequent follow-up appointments.

## Discussion

Due to the variability of symptoms and the absence of clear diagnostic criteria, nutcracker syndrome is a common underdiagnosed condition. Based on de Schepper's study, the nutcracker syndrome is defined as the associated symptoms with compression of the left renal vein between the superior mesenteric artery and the aorta [4]. Many reports show that it is more prevalent in females and common in the second to fourth decades of life [5-7]. Symptoms vary from asymptomatic microhematuria to severe pelvic congestion though some patients have severe and persistent symptoms such as pelvic pain, flank pain, and gonadal varices aggravated by physical activity and orthostatic intolerance [5,8]. Nonetheless, in one systemic study, the absence of hematuria in nutcracker syndrome is about 20% [9].

It is reported that 83% of patients with nutcracker syndrome suffer from pelvic congestion due to pressure on the left renal vein from compression by the aorta and the superior mesenteric artery [10]. The diagnosis of this condition is based on a stepwise workup with history and physical examination, followed by Doppler US, CT, MRI, and intravascular US [8].

Some CT scan findings are reported to be practical steps in diagnosing the nutcracker syndrome. The beak sign which is due to the abrupt narrowing of the left renal vein because of compression between the superior mesenteric artery and the aorta helps diagnose nutcracker syndrome [11]. One retrospective study also demonstrates that a pre-compression to compression ratio of the left renal vein is over 2.25 and has 91% specificity and sensitivity to diagnose nutcracker syndrome [12]. The compressed segment was defined as the diameter of the left renal vein at the point of maximal compression between the superior mesenteric artery and aorta, and the pre-compressed segment was measured at the uncompressed segment diameter of the left renal vein proximal to the passing between the aorta and superior mesenteric artery.

Moreover, Takebayashi et al. suggested that using the color doppler sonography and correlating it with flow patterns on retrograde left renal venography would add value to diagnosing nutcracker syndrome noninvasively [13]. However, due to the particular circumstances that our patient was under, this was not performed.

In terms of management, it is still controversial, and there are different opinions based on the symptoms and severity from observation to invasive interventions such as open surgical procedures, intra or extravascular stents, and intrapelvic chemical cauterization. However, the consensus is for young and mildly symptomatic patients; an observation approach is acceptable whilst for patients more than 18 years old with severe symptoms such as intolerable flank pain, impairment of renal function including persistent orthostatic proteinuria, varicocele formation, gross hematuria, or failure of observation or medical treatment, invasive treatment is recommended [5,6,8]. In one case report, angiotensin-converting enzyme inhibitors had shown improvement in orthostatic proteinuria with nutcracker syndrome [14].

## Conclusions

Nutcracker syndrome accompanying pelvic congestion syndrome is a rare pathology that can be easily missed unless there is an understanding of the syndrome and proper assessments are done as in our case where the patient needed repeated CT scans. It should be considered in the differential diagnosis of patients with nonspecific abdominal pain, flank pain, or hematuria, especially in female patients. The diagnosis of nutcracker syndrome involves a combination of clinical assessments, laboratory studies, and radiological investigations. Hence, there is a greater need for dissemination and understanding of the syndrome and its clinical variants among the main specialties involved to optimize patient care.
